# Usability testing of a digital tool to support behavioural activation for depression with young people aged 12 to 18

**DOI:** 10.3389/frcha.2026.1799929

**Published:** 2026-05-21

**Authors:** Lucy Tindall, Philip Kerrigan, Emma Standley, Susan Metcalfe, Matthew O’Brien, Lina Gega

**Affiliations:** 1 Department of Health Sciences, University of York, York, United Kingdom; 2 Research and Development, Tees Esk and Wear Valleys NHS Foundation Trust, Durham, United Kingdom; 3 Hull York Medical School, University of York, York, United Kingdom

**Keywords:** behavioural activation, depression, digital interventions, template analysis, young people

## Abstract

**Background:**

Adolescents with depression cannot access timely support due to pressures on specialist mental health services. We developed and tested a manualised version of behavioural activation—a brief psychological intervention—for delivery in the community by non-specialists. We digitalised the paper version of the behavioural activation manual as a more accessible and user-friendly route for completing therapy tasks.

**Methods:**

Young people who received manual-based behavioural activation as part of a large trial were invited to test the digital tool. Following a demonstration, participants were asked to use the digital tool in their own time over 4 weeks, completing tasks such as activity scheduling that they had previously completed using pen and paper. Participants then attended a one-to-one interview lasting approximately 30 min to provide feedback on the digital tool. All interviews were audio-recorded and transcribed verbatim and analysed using Template Analysis.

**Results:**

Overall, 14 participants, aged 13-to-17-years *[M:*15.4 (*SD* = 1.6)] and comprising 10 females, 3 males and one individual who was non-binary took part. Five key themes, each comprising two subthemes, were identified. These centred on a) the perceived strengths of the digital tool, b) access and engagement, c) using the digital tool both independently and with a professional as well as its position in the wider treatment pathway, d) the role of the digital tool in relapse prevention and e) suggested ways to enhance the overall utility of and engagement with the digital tool.

**Conclusions:**

Overall, the young people perceived the digital tool to be a useful and accessible resource that could support their mental health journey, both during and after therapy. The digital tool's features for planning, reflection, and visualizing progress were particularly valued. Identified improvements included the incorporation of reminders, in-app guidance, customization options, and a journalling feature to enhance engagement and the therapeutic value of the digital tool.

**Clinical Trial Registration:**

https://doi.org/10.1186/ISRCTN57377955 Identifier: ISRCTN57377955.

## 1 Introduction

Globally one in seven 10–19-year-olds experience a mental disorder, accounting for 15% of the burden of disease in this age group (1). In the UK, where this study is based, approximately 20% of 8-to-16-year-olds and 23% of 17-to-19-year-olds have a mental health condition (2) with depression the most commonly experienced. Poor mental health in young people is associated with social withdrawal and lower levels of school attendance and educational attainment, especially where there is more than one concurrent mental health problem or where these occur alongside behavioural problems (3). If not treated effectively and promptly, mental health challenges, including depression, can persist into adulthood with knock-on individual and societal-level impacts including reduced quality of life, increased healthcare costs and reduced economic productivity. Many young people cannot access appropriate support because specialist services commonly have long waiting lists and high entry thresholds (4) and it is both an international and UK government priority to identify and roll-out new economically sustainable interventions, including solutions that leverage digital capabilities (5).

In 2021 we initiated a large programme of research called ComBAT (Community-Based Behavioural Activation Training for Depression in Adolescents), to develop and test a new community-delivered Behavioural Activation (BA) to support young people aged 12–18 with mild-to-moderate depression. BA is an evidence-based psychological intervention which aims to restore and increase stable sources of positive reinforcement in a person's environment through purposeful and rewarding activities. We selected BA as the basis for our intervention as it can be delivered by non-clinicians in non-clinical settings (6, 7) and requires fewer sessions and shorter training than alternative approaches (e.g., Cognitive Behavioural Therapy), making it less resource-intensive. As part of ComBAT, we found a strong appetite for a digital tool that would support the completion of BA. Specifically in facilitating tasks including identifying value-aligned activities and participating in activity scheduling/scoring.

Alongside ComBAT we conducted a systematic review to explore the existing evidence on the effectiveness of BA for children and adolescents with depression. Of the studies we identified, two reported on the delivery of BA to young people in a wholly digital format (8), reporting the approach to be feasible and acceptable (9, 10). Neither of these existing digital tools however incorporated all the functionality needed to properly support our BA. Firstly, we needed a digital tool that could be used initially alongside sessions with a professional therapist. Secondly, whilst they contained a number of ingredients of BA, including activity scheduling, they did not incorporate a value-based approach, that is identifying activities which align with what is important to the young people in different areas of their life (e.g., relating to their hobbies, their family and friends). We separately identified an additional BA-based app for adults—the “Moodivate” app (11). Whilst this did include a values-based component, it was designed primarily for adults and was not entirely compatible with our BA as it provided no support for individual activity scoring. We therefore developed a new, bespoke prototype digital tool that is accessible by web-browser on a phone, computer or tablet. This article reports on the usability testing of this first version of the digital tool to gather feedback from young people and suggestions for refinement.

### 1.1 Aims and objectives

This study aimed to test the first version of the newly developed BA digital tool and gather feedback from users to inform its further development. Specifically we sought to gather the following information:

1. What were young people's perceptions of the digital tool overall? (What did they like/ not like, what did they find useful etc.)
2. How did young people feel the digital tool would best fit within current BA delivery and about its continued use after sessions have finished?
3. How did young people feel the digital tool needed to be adapted/extended to increase its utility and enhance engagement?

## 2 Materials and methods

### 2.1 Design, settings and participants

This usability study, involving qualitative interviews and embedded within a Randomised Controlled Trial (RCT) (https://www.ComBATdepression.org), was conducted between November 2023 and June 2025 across a range of NHS and community-based sites including schools, colleges and charitable organisations. Potential participants were 12-to-18-year-olds who had taken part in the ComBAT RCT and received a minimum of 5 sessions of manualised BA. Eligibility for the RCT was defined by a T-score of ≥65 on the depression subscale (10-items) of the Brief Revised Children's Anxiety and Depression Scale [RCADS-25 (12]) and a score of <15 on the Patient Health Questionnaire-9 items modified for adolescents [PHQ-9A (13)]. The RCADS-25 was our primary outcome measure and was selected because it is a nationally recommended outcome measure as part of the Mental Health Services Dataset (MHSDS) in the UK and is routinely collected by professionals within clinical services with children and young people. Inclusion of the PHQ-9A helped us to screen out those with severe presentations which were beyond the remit of the trial. (For further methodological details see https://www.isrctn.com/ISRCTN57377955).

### 2.2 Procedure

The researcher who conducted the final follow-up at 12 months post-randomisation gave written and verbal information about this study to participants who consented to receiving information about further studies on enrolment to ComBAT. For those who expressed an interest, a meeting with a clinical member of the research team was arranged online or face-to-face, to suit the participant. At this meeting, informed consent from the young person (if ≥16 years) or consent from the parent and assent from the young person (if ≤15 years) was obtained. Consented participants were given a brief overview of how to use the digital tool and then asked to test it out in their own time over four weeks. Participants could choose how and when to use the digital tool with no expectations as to usage and no usage data collected. Although the main ComBAT intervention is delivered over 5–8 sessions (preferably delivered weekly), four weeks was deemed appropriate for testing the digital tool as (i) the young people were already familiar with the intervention and (ii) the digital tool contains considerably less content than the original ComBAT manual.

A research team member contacted participants two weeks after they had been given digital tool access to check progress and to answer any questions. After four weeks participants attended an individual interview lasting 15–30 min where they discussed their experience of using the digital tool. Interviews followed a structured topic guide and were audio recorded and transcribed verbatim. Participants received a £25 voucher at the start and a further £25 voucher after their interview in recognition of their contribution to the research.

### 2.3 Intervention development and content

Having consulted the published literature and liaised with key stakeholder groups (young people, parents/guardians, professionals), we developed our ComBAT BA for delivery within community-based settings. The intervention is organised into 5 “modules” which can be completed in 5–8 weekly sessions of 30–45 min each in a blended model of professional-guided sessions and self-directed activities. The intervention was initially tested in a feasibility study (14) in which three-quarters of *n* = 20 participants demonstrated a “positive” change in scores on the RCADS from baseline to follow-up. A subsequent RCT is currently in the analysis phase and will be published upon completion. Whilst the original version of the intervention was completed using a paper-based manual, the digital tool enables users to complete key elements of BA activities via an online interface instead. Specifically, the digital tool has three key functions: (1) Users identify activities they value that relate to different areas of their life—“my people, my world, myself, other” and add these to an “activity bank”. (2) They schedule activities from this bank into a personal calendar. (3) They review the activities they have undertaken and rate them according to the emotional rewards “pleasure, achievement, sense of connection” they derived. Alongside these key features is an analytical toolkit which provides easy to understand visual summaries of the type of activities carried out over a chosen period. These summaries are automatically computed and introduced to encourage continuous engagement with BA (during and more importantly after working with professional therapists). (See Supplementary A for graphics of the digital tool functions).

### 2.4 Sample size

There is limited guidance available regarding the optimum sample sizes for studies of this design. However Wiklund et al. (15) proposed that the minimum sample for a summative evaluation is 15. Therefore, we provided study information to all young participants who met the eligibility criteria (see 2.1) and agreed to be contacted about further research. The sample size was deemed sufficient to achieve diversity and depth in interview responses. Those assenting/consenting entered the study until a sample of 15 had been attained. No individuals who met the inclusion criteria were excluded.

### 2.5 Data analysis

All interview transcripts were analysed using Template Analysis (16). As no usage data was collected all transcripts were analysed collectively, irrespective of how much each participant used the digital tool. This allows for both deductive and inductive coding by requiring the definition of a number of *a priori* themes which are then evaluated for their appropriateness and completeness against a subset of transcripts with codes modified and/or added as needed so as to better thematically organise and describe the data. We started our analyses with 7 *a priori* themes which had been included in our topic guides (accessibility, content and functionality, engagement, overall impressions, presentation, relapse prevention and user experience). These topic guides were developed in consultation with our ComBAT study expert-by-experience and patient and public involvement and engagement representatives. Independently, two researchers coded a small subset of transcripts (*n* = 3) against the *a priori* themes and produced an initial template. Following several modifications, a final template was produced against which the remaining transcripts were coded and interpreted. The two researchers met to resolve any discrepancies in order to increase the reflexivity of the process and reduce the subjectiveness of the framework. Had any disagreements occurred, then a third member of the research team would have been consulted.

## 3 Results

### 3.1 Uptake, completion and follow-Up

Overall, 36 young people were given information about the study at their ComBAT RCT 12-month follow-up with 21 returning an expression of interest form. Of these, 15 consented/assented to participate whilst 6 changed their minds or became non-contactable. Fourteen participants had access to the digital tool over four weeks and provided feedback via an individual interview. The remaining participant became uncontactable and was therefore withdrawn.

### 3.2 Participant characteristics

The fourteen participants ranged from 13-to-17-years (*M* = 15.4 (*SD* = 1.6) and the sample comprised 10 females, 3 males and 1 non-binary young person. Most participants (*n* = 11) were English/Welsh/Scottish/Northern Irish/British with the remainder from other mixed/multiple ethnic backgrounds (*n* = 1), Gypsy or Irish traveller backgrounds (*n* = 1) or White and Black African backgrounds (*n* = 1). The participants were predominantly in secondary (*n* = 8), or higher education (*n* = 5) with only one participant not in education. Six participants were atheist, 3 (Catholic, 2 agnostic, 2 Protestant (Church of England) and 1 (“Other Christian”). The participant demographics are presented in Table 1.

**Table 1:** Participant characteristics (*n* = 14).

ID	Gender	Age^a^	Ethnicity	Current education status	Religion	Number of BA sessions completed
A	Female	16	English/Welsh/Scottish/Northern Irish/British	Not in education	Catholic	9
B	Female	17	English/Welsh/Scottish/Northern Irish/British	Further/Higher	Catholic	6
C	Male	16	Any other mixed/multiple ethnic background	Further/Higher	Atheist	8
D	Male	17	English/Welsh/Scottish/Northern Irish/British	Further/Higher	Other Christian	7
E	Female	16	Gypsy or Irish Traveller	Secondary	Atheist	6
F	Non-binary	13	English/Welsh/Scottish/Northern Irish/British	Secondary	Catholic	7
G	Female	17	English/Welsh/Scottish/Northern Irish/British	Secondary	Agnostic	8
H	Female	15	English/Welsh/Scottish/Northern Irish/British	Secondary	Protestant (Church of England)	9
I	Female	16	English/Welsh/Scottish/Northern Irish/British	Further/Higher	Atheist	7
J	Female	13	English/Welsh/Scottish/Northern Irish/British	Secondary	Atheist	6
K	Female	16	English/Welsh/Scottish/Northern Irish/British	Secondary	Agnostic	8
L	Male	13	English/Welsh/Scottish/Northern Irish/British	Secondary	Atheist	12
M	Female	13	English/Welsh/Scottish/Northern Irish/British	Secondary	Atheist	7
N	Female	17	White and Black African	Further/Higher	Protestant (Church of England)	6

a Age at time of interview.

### 3.3 Key themes

**Figure 1:**
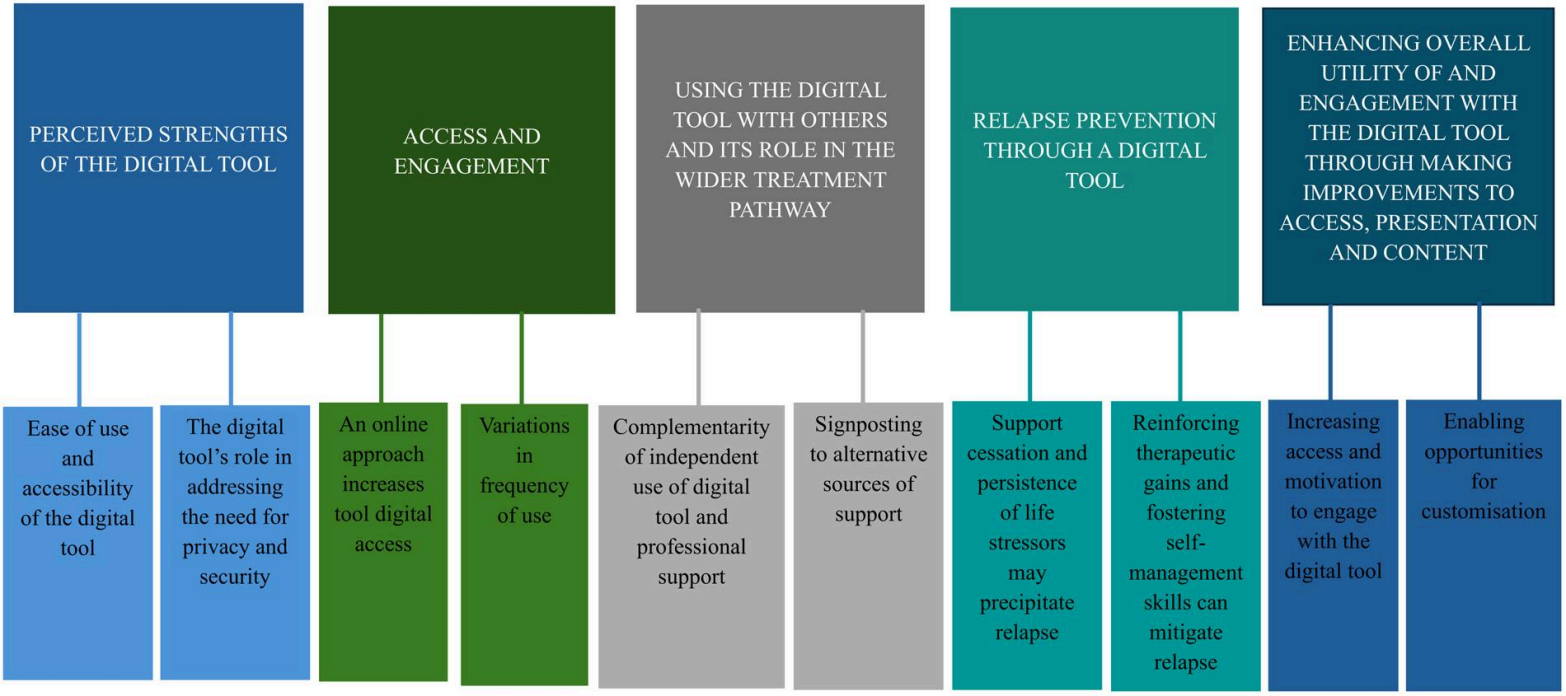
Coding hierarchy.

Overall five overarching themes, each comprising two subthemes, were identified from the interviews with young people when discussing their use of the digital tool. These centre on perceived strengths of the digital tool, access and engagement, using the digital tool both independently and with a professional as well as its position in the wider treatment pathway, the digital tool's role in relapse prevention and suggested ways to enhance the overall utility of and engagement with the digital tool. The themes and subthemes are presented in Figure 1.

#### 3.3.1 Theme 1: perceived strengths of the digital tool

##### 3.3.1.1 Sub-theme 1.1: ease of use and accessibility of the digital tool

The digital tool was widely praised for its convenience and accessibility. It was regarded as easy to use, helpful and clearly presented.

“It is a really good app, it's very helpful, it helps you keep track of things” [Participant E]

The young people were overwhelmingly positive when discussing the digital tool's interface. Overall, the digital tool was found to be “clear”, “simple”, “well-organised” and “easy-to-use” with several users stating that they would not make any major changes to the layout. Most users found it easy to navigate through the digital tool's features and input information.

“It literally tells you what to put and where. So, it's pretty … it's, like, simple.” [Participant B]

“It's really well designed, so it's like basically within ten minutes of looking at it, you know where everything is.” [Participant C]

Some however, needed to use the digital tool for a few sessions before they completely understood how it worked:

“I did need to use it one or two times before I could … before I fully, like, understood what everything did and where everything was. But now that I've used it, it's … I do think it's very easy to use.” [Participant L]

Two young people described finding the homepage initially a little confusing until they had clicked through the content:

“I think the first page, like the homepage, when you look at it, it can look a little bit confusing until you click into each section”. [Participant N]

Generally, the young people liked how the digital tool was presented; several stated that the colour scheme made the digital tool feel “cheery” and engaging whilst the use of pictures and diagrams were seen to provide helpful visual aids:

“Then it's got loads of colours and little diagrams on it and I think that's a good thing because it, kind of, makes you want to stay on the app, because you don't think it's boring. Because if it was, like, in black and white with no diagrams or anything, you're going to be like oh, well I don't want to look at this anymore, I'm bored”. [Participant E]

The digital tool's design successfully incorporated several additional elements to the manual and worksheets that facilitated self-reflection. The most notable of these being the graphs showing activity engagement plus activity score changes over time. These were consistently praised for presenting information in an accessible, easy-to-read format.

“when there's some sort of visual, it's a lot easier to figure out what you're actually reading”. [Participant E]

##### 3.3.1.2 Sub-theme 1.2: the digital tool’s role in addressing the need for privacy and security

Another key benefit identified was enhanced privacy. Participants felt more secure using a password-protected digital tool on their phone rather than a physical booklet. This was seen as a major advantage, particularly for those who might want to conceal their mental health struggles.

“And it's more privacy as well “cause if you’ve got it written out in paper, someone can find it, do you know what I mean, it's just it's on your phone, it's locked, if you’ve got a password on your phone, it's locked away, no one can find it. And I just think it's really beneficial for a lot of people”. [Participant B]

Several participants also noted that the digital tool was easier to keep safe and track of than paper-based materials, which were often forgotten, lost, or damaged.

“I often forgot, like, the piece of paper at home, or I’d lose it or something, and I’d need a new one. But with the online resource, I think I’d remember more often.” [Participant A]

#### 3.3.2 Theme 2: access and engagement

##### 3.3.2.1 Sub-theme 2.1: an online approach increases digital tool access

Participants consistently highlighted that the online format made it easier to access and use than physical resources:

“it was easy to get onto, and also you can go back any time, because it's online, so you don't have to look through papers”. [Participant A]

The ability to use the digital tool on a phone was a major advantage. Those accessing it this way valued the portability which allowed them to record activities and thoughts “in the moment”, rather than having to remember to do so later. Some noted that the digital format felt “less overwhelming” than paper.

Participants described the digital tool as “something there for them all the time”, providing a sense of continuous support. This was particularly true for those who accessed the digital tool on their phone with one young person highlighting that as they always had their phone with them they had continuous access to the digital tool. Specifically, individuals could quickly look back at any point to see how undertaking an activity “made you feel” and to reflect on progress. In this sense the digital tool was a powerful motivator, increasing activity and mood.

“I also use it to see how much I’ve improved from what I was and then that helps me get in a better mood” [Participant B].

While the young person could potentially achieve the same result from looking back at the pen-and-paper worksheets, the digital tool makes this process much easier, with all the information stored and ordered together digitally with “everything in one place”. Participants who disliked writing by hand or had difficulty reading their own handwriting found the digital format particularly helpful:

“I don't really like writing by hand and sometimes I can find it difficult to read my own handwriting. So being able to do it on, like, my phone where I don't need to try and struggle to read my handwriting would help me a lot. But also, I can use it wherever I go, I don't need to bring the sheets with me”. [Participant L]

##### 3.3.2.2 Sub-theme 2.2: variations in frequency of use

Participants' self-reported use of the digital tool varied, with some indicating frequent, near-daily engagement. Several participants who used the digital tool consistently linked this to the establishment of a routine:

“I think I was using it pretty much every day, if I got myself into the routine. ‘Cause it really helped me, like, the next day, setting up tasks.” [Participant J]

However, many participants described a less consistent pattern of use. Reported frequencies ranged from “five or six times” over the course of four weeks to two or three times per week. For these individuals, engagement was often dependent on memory or triggered by a specific event or task they wanted to schedule.

Another participant candidly described a pattern of remembering and forgetting, stating they used it for one day, forgot the next, remembered the day after, and “never did it again.”

Overall, the flexibility of the digital tool, with young people able to choose when to use it, was appreciated.

“Yeah, and there's no pressure to, like, continuously use it, you can just use it, like … ’cause that's the other thing, sometimes if you have to do something everyday, it can be quite a lot if you’re not feeling the best”. [Participant H]

#### 3.3.3 Theme 3: using the digital tool with others and its role in the wider treatment pathway

##### 3.3.3.1 Sub-theme 3.1: complementarity of independent use of the digital tool and professional support

All users received a guided introduction from a clinician before using the digital tool. Several young people highlighted the importance of this in allowing them to fully understand the digital tool's features and capabilities and in successfully going on to use it independently:

“He showed me how to actually get onto it … we went through the App before I left him, so I knew, sort of, what everything was for … Sorry, that definitely helped.” [Participant H]

For future versions, it was suggested that an introductory video explaining how to use the digital tool would be a helpful addition.

“Yeah, definitely. Even if it was just, like, a video just, kind of, explaining, like, this is what this is for, how to write on it and stuff, ‘cause I didn't realise some of the stuff you could click on and actually write on”. [Participant H]

Experiences of the participants going on to use the digital tool independently during the user-test period were mixed. Some expressed positive feelings about using the digital tool on their own terms, enjoying exercising autonomy and taking personal responsibility for their progress:

“It was the, like, independence of just using it myself that I liked.” [Participant L]

Despite this, some also found it challenging to self-motivate to use the digital tool in the absence of continuing professional engagement and support:

“‘Cause I wasn’t talking to someone weekly, it was, kind of, drifting away from me, like remembering to actually go on it.” [Participant-H]

As a result, a consensus emerged that the digital tool would be best used in practice as part of a blended treatment approach where it complemented, rather than replaced, professional support:

“I think it should be used alongside the therapist … ” [Participant F]

“So, I think, with that, it would be good to using them both at the same time, and then being able to bring what you get from the app to your session and slowly you can purely go up, doing it by yourself on the digital version, but I guess, some way of maybe organising sessions through the app, or if you think you need to go back to seeing someone, you can do it via the digital version.” [Participant C]

Key suggestions for the role professionals could play included providing a demonstration of how to use the digital tool and supporting the completion of more complex activities like goal setting.

Some participants further saw the digital tool as a way to bridge the gap between clinical sessions and daily life and enhance their communication with therapists. Sharing the data on activities and mood tracked by the digital tool could give the therapist another way of monitoring progress which would be particularly useful if a young person was finding it difficult to verbalize their feelings in a session. Alternatively, it could provide a more objective and detailed account than memory alone might allow.

“Because kids can sit there and just say, yeah, everything's fine, when therapists have got the option to have a look and they can go, hang on we know you’ve had a bit of a rough day, is there anything going off there, kind of thing?” [Participant F]

This suggests the digital tool could function as a “third party” in the therapeutic alliance, a shared data point that facilitates deeper and more focused conversations.

##### 3.3.3.2 Subtheme 3.2: signposting to alternative sources of support

The desire for additional support and guidance embedded within the digital tool was also discussed. Suggestions included providing the telephone numbers of support services (e.g., children's mental health charities) in case young people needed additional support and providing self-help tips. Some also suggested setting up a “community section” where users could talk to others who have shared similar experiences.

“I feel like having just something, a page of, like, people you can go and talk to. I’ve forgot the names of them. But just a list of people you can go and talk to and maybe some sort of page with distractions and stuff and, like tips on how to calm down and just distract yourself”. [Participant E]

“They could have, like, not definitions, but like, things to guide you through it. And then also maybe, like, numbers in case you did need help” [Participant A]

#### 3.3.4 Theme 4: relapse prevention through a digital tool

##### 3.3.4.1 Sub-theme 4.1: Support cessation and persistence of life stressors may precipitate relapse

Several young people described their perceptions of relapse risk following the conclusion of BA. Participants primarily attributed the potential for relapse to the cessation of support and the persistence of life stressors without a dedicated outlet for processing them.

A primary factor identified by participants was the abrupt withdrawal of consistent, professional support. The end of therapy was described as a significant “big change in the routine” that removed a crucial, reliable space for young people to “offload any problems.” One participant articulated this starkly:

“you see someone once a week, and then it just stops.” [Participant A]

Another participant echoed this, suggesting that after support is withdrawn, individuals may “slowly just … deteriorate to where they were before” because they have “lost that support.” One participant articulated a common sentiment:

“Some people just need something there for them all the time”. [Participant B]

Without this ongoing support structure, there is a risk of losing the progress made during treatment. This is particularly so because ongoing life pressures, such as from school, continue after therapy ends. Without a therapeutic outlet however, these stressors can become overwhelming.

##### 3.3.4.2 Sub-theme 4.2: reinforcing therapeutic gains and fostering self-management skills can mitigate relapse

Participants identified several ways in which the digital tool could actively help mitigate relapse risk, after sessions with the therapist have finished, by reinforcing therapeutic gains and fostering self-management skills.

The digital tool was valued as a personal resource with young people describing how they had used it to revisit strategies that they had previously found effective:

“And it's just something, like, it's just nice, like, you can go back to it and it's, like, no matter how I’m feeling, like, even if I’m having a good day I’ll flip back and I’m, like, oh I can do this to make me feel even better” [Participant B]

This ability to access personalized coping mechanisms on-demand was seen as a powerful feature for managing difficult moments.

The digital tool's capacity to log activities and moods allowed users to continue to reflect on positive changes in their mood post-sessions. This process of reviewing personal progress was described as a mood-enhancing activity in itself, reinforcing a sense of achievement and personal growth, which is a key component in preventing relapse.

“I'd say probably having the encouragement of knowing if the sessions are finished … I feel like having the encouragement and the reassurance of knowing that they can still be able to express what they're feeling just in a different way.” [Participant C]

“It's just nice to have something to reflect on, like, so you’re, like, and I also use it to see how much I’ve improved from what I was and then that helps me get in a better mood ‘cause I’m, like, I was really bad at this point and I’m not there anymore”. [Participant B]

Features such as goal setting and daily planning were used to maintain a sense of routine and purpose. One participant found it “easier to plan my stuff with it” [Participant B], demonstrating the digital tool's utility in structuring daily life, an area that can be challenging for those recovering from depression.

Participants saw a key role for the digital tool as a stepping stone from formal therapy to self-management. It supports them through a structured and familiar framework to continue to practice BA after sessions with the therapist have ended, thereby helping them maintain progress long-term—to “actively try and keep up with your mental health” [Participant J].

#### 3.3.5 Theme 5: enhancing overall utility of and engagement with the digital tool through making improvements to access, presentation and content

Although the feedback was overwhelmingly positive, the participants identified some areas for future improvement that would enhance the overall utility of and engagement with the digital tool. Suggestions particularly related to increasing access and allowing opportunities for customisation.

##### 3.3.5.1 Sub-theme 5.1: Increasing access and motivation to engage with the digital tool

In the digital tool's current format young people accessed it by following a link and entering their log in details however some couldn't remember this information, misplaced the link thus losing access or frequently forgot to access it:

“Cause quite a few times I got a bit, like, lost in the email and then didn't know how to actually get onto it. Or, like, I couldn't remember what it was written down as”. [Participant H]

The digital tool relied on a Google account for login which was also flagged as a potential barrier. Participants suggested alternative sign-in options like iCloud or phone numbers to make it more inclusive. Most suggested developing the digital tool as a dedicated app rather than a website. An app, they proposed, would be more reliable, remain logged in, and be easier to find on a phone's home screen, which would help them remember to use it regularly.

“Well it would be nice if everyone had it generally, but if you have it on the app, then you’ve always got it with you. So, if you ever feel like something's going to happen, you can go on the app, look at some tips and talk to someone if you need to”. [Participant E]

In addition, one suggested that access to the digital tool would be improved if it were integrated into an existing digital ecosystem:

“if you’ve got it in a folder with, like, Instagram, whatever you use more frequently, you’re going to be able to go to it rather than having to go to Google and search for the website” [Participant N].

To promote use of the digital tool, participants suggested advertising it on platforms like Instagram or TikTok, and having mental health support staff recommending it in school mental health sessions. Another suggestion was including information about the digital tool in existing mental health leaflets.

Several participants suggested that the absence of reminders and notifications contributed to low engagement, and emphasised the need for regular prompts to encourage consistent digital tool use.

“But it's very easy to forget about because nothing … like, you get no reminders, no pop-ups and I feel like if it had more pop-ups, I would have used it more.” [Participant E]

Young people discussed how reminders could take the form of daily prompts to log in or track and/or review activities. One user suggested that these notifications should be framed carefully, in an empathetic way and avoiding an accusatory tone. Instead of “you haven't been on today,” an alternative approach could be “has it been a hard day, do you want to talk about it?” to prompt reflection without inducing guilt. Similarly, participants suggested the possibility of adding in simple motivational quotes:

“[L]ike, telling you every day that it's going to get better” [Participant A]

Several participants suggested introducing an element of gamification to interactions with the digital tool. They cited popular apps, including Duolingo, proposing that incorporating a “streak” feature would motivate continued engagement:

“So, I think some, kind of, daily reminder or something similar to, like, a Duolingo streak, the thing that would keep people going back to it and keeping using it”.[Participant C]

Another gamification feature mentioned was that employed in the “Forest” app where users are rewarded for focusing on their work by a seedling they plant growing into a tree. A young person described how this had given them a sense of accomplishment and they therefore felt that the digital tool could benefit from something similar.

Several suggestions were made for functional enhancements to improve the digital tool's utility and support its therapeutic goals. Adding a dedicated space for free-text journaling or note-taking where users could record any thoughts and feelings during digital tool use was frequently suggested. Participants wanted to add comments to their activity reviews to provide context to their mood scores which they felt would be helpful when reviewing and reflecting on completed tasks. In addition, users wanted more flexibility in how they logged activities. Suggestions included allowing recurring entries for routine activities, the ability to add specific one-off events directly to the calendar without cluttering the main activity log, and options for multiple-choice selections when categorizing an activity (e.g., doing something with both “family and friends”).

To further support the usability of the digital tool it was suggested adding definitions of key terms to aid understanding and including a help section containing specific information on how to navigate the digital tool.

##### 3.3.5.2 Sub-theme 5.2: Enabling opportunities for customisation

Several participants discussed the importance of adding opportunities for customisation, particularly relating to visual elements of the digital tool. The ability to change background colours was suggested as was allowing users to select a “dark mode” to present the digital tool's content. This was proposed to both support those who may struggle to read text on a white background (e.g., those with dyslexia) and to reduce eye strain:

“The only thing, I wouldn't mind a dark mode with it just because, at times, my eyes … I get eye strain because it is quite bright, but that's pretty minor” [Participant C]

“I struggle with reading texts that are black or white. So, I feel, like, a coloured background would be helpful”. [Participant J]

## 4 Discussion

### 4.1 Summary of findings

In this study we gathered feedback from fourteen young people (aged 12-to-17-years) about a newly developed digital tool to support the delivery of BA for low mood/depression. This digital tool was designed to be used both within sessions with a professional and independently when treatment sessions have ended. Overall we identified five key themes, each comprising two subthemes. These specifically focused upon (1) perceived strengths of the digital tool, (2) access and engagement, (3) using the digital tool both independently and with professional support as well as its position in the wider treatment pathway, (4) the role of the digital tool in relapse prevention and (5) suggested ways to enhance the overall utility of and engagement with the digital tool.

Our first objective was to explore what young people's perceptions of the digital tool were overall. On the whole, participant feedback was positive with users particularly valuing the online approach as allowing for increased privacy, accessibility and flexibility of use. Most users found the digital tool easy to use and navigate and described the presentation and graphics as engaging. They particularly appreciated the opportunity to track the progression in their activity scores via the graphs. They also liked the flexibility of use supported by the digital tool, with some using it daily and others less frequently, but with it always being available and accessible if needed.

Our second objective was to explore how young people felt the digital tool would best fit within current BA delivery and how it might continue to be used after sessions have finished. Several participants felt that the digital tool would be best placed within a blended treatment format, that is with concurrent support from a professional. This would help to pre-empt any difficulties with self-motivation that might arise were the tool to be positioned from the start more as a self-managed resource. Participants described the digital tool as having the potential to bridge the gap between therapy sessions by supporting the continuing integration of BA into day-to-day life. In the same vein, they liked how the information logged in the digital tool could be used to update the professional about what they had been doing if they were struggling to verbalise this. They also saw an important role of the digital tool as aiding the transition from therapy to self-management and helping to maintain practice of BA, sustain mood and prevent relapse. It was evident from their responses that these twin functions—of supporting engagement with BA during and after seeing the therapist—were viewed as complementary and of equal importance.

Our third objective was to identify the ways in which young people feel the digital tool needs to be amended, specifically in supporting its autonomous use after BA sessions have ended. To address issues with self-motivation and enhance engagement with the digital tool a number of refinements were suggested. Specifically, simplifying the methods for accessing the digital tool and, embedding reminders to encourage regular use, elements of gamification and adding space for free-text to the activity reviewing. Young people also suggested users being able to customise the visual elements of the digital tool to accommodate individual needs and increase engagement.

### 4.2 Our findings in the context of current literature

Overall our research has demonstrated that, for many young people with less severe presentations of depression, a digital tool could both support the delivery of BA via standardised sessions with a professional and also provide a scaffold for the transition to self-management and continuing autonomous use of ComBAT BA once professional contact ends. These findings build on those of two previous studies of online BA (9, 10) identified in our 2024 systematic review which demonstrated a digital approach to be both feasible and acceptable (8). There is a significant difference however in that the online tools in both these studies were self-guided rather than used (initially) alongside face-to-face support from a professional therapist. Although these two earlier studies suggested that a self-guided approach was feasible and acceptable, several of our participants strongly expressed the view that our digital tool would be best situated within a blended treatment format. Although there is currently no evidence on the effectiveness of BA delivered in this blended format, there is an ongoing RCT investigating the effectiveness of blended BA for young people with depression in specialist child and adolescent mental health services (BAY: https://doi.org/10.1186/ISRCTN12315118). There are also several systematic reviews and meta-analyses demonstrating that closely related, CBT-based, interventions for young people with common mental health disorders and which comprised both a digital and an in-person element (i.e., therapist, parent, and peer), resulted in greater adherence and were more effective than those that were fully automatized or self-administered (17).

The participants cited several benefits over pen and paper , including more convenient access—they carry their phone on them nearly all the time—and offering a more appealing way of completing the BA elements, including the calendar, scoring etc. These benefits imply that a digital tool would improve the likelihood of young people engaging with BA between sessions as well as the quality of that engagement. There is indeed evidence from other studies of digital tools promoting completion of homework or between session assignments in mental health interventions for young people (18). Our finding that users particularly valued the online approach in allowing opportunities for increased privacy, accessibility and flexibility of use; is also concordant with previous research (19, 20), as is our finding that young people want opportunities for customisation to accommodate individual needs and increase their engagement (17). All of these principles fit squarely too within the “Persuasive Systems Design” framework, identified in a recent article reviewing 103 mental health apps (21).

It is generally agreed that there is a significant challenge in sustaining the progress made following an intervention and reducing the risk of relapse (22). Our young person participants supported this view with several describing the potential negative impact from the “abrupt” disappearance of support at the end of sessions with a professional. They identified several potential new features, and refinements to existing features, to add to the digital tool that could mitigate this and offer support for continued engagement and reduced risk of relapse. One of these suggestions, automated reminders, has been shown to increase engagement and effectiveness in a number of studies (23, 24).

### 4.3 Strengths and limitations

Recruiting our overall target showed that young people were happy to use the digital tool and provide feedback on its use. Furthermore, the participants were recruited from 7 different sites representing variation in site type (i.e., school/college, NHS service, charitable/voluntary organisation) and geographical location. The digital tool was professionally created and intentionally designed to reflect the ComBAT paper manual, allowing consistency and familiarity for the participants.

Despite this, the study had several notable limitations, meaning our results need to be interpreted with caution. Most (10/14) of our sample identified as female, limiting the transferability of our findings to a more gender-diverse population. In addition, all participants had received at least 5 sessions of BA with a supporting professional and thus the sample may have been biased towards individuals more likely to engage with treatment. Whilst this meant that the sample was well placed to provide feedback on the digital tool, this may have negatively impacted upon the transferability of the findings. In particular, transferability to those who disengage from support early and individuals who are less digitally confident. Gathering the opinions of some young people such as these, might have allowed insights into components of the digital tool needed to maintain/increase engagement.

Aligning to the timelines of the digital tool development and within the remit of the study, participants were only given access to the digital tool after sessions with a professional had ended. As a result we were unable to gather feedback pertaining to using the digital tool during BA sessions with a professional as well as post-treatment. Future research will seek to explore this and also gather the views from supporting professionals as well as young people.

### 4.4 Recommendations for intervention refinement and future evaluation

Based on the experiences and feedback of our participants, we will adapt and develop the digital tool in a number of ways. These changes aim to increase engagement with a future version of the digital tool through improvements to access, presentation and content. The following additions will be made:

- Alternative sign-in options through e.g., iCloud or phone numbers
- Notifications/reminders/prompts/motivational quotes, ensuring these are empathetic in tone
- Gamification elements, such as “streaks” or rewards for engagement and achievement of activities
- A section for free text journalling/note-taking particularly for users to add comments to their activity reviews
- Flexibility in logging activities—e.g., recurring activities and options for multiple-choice selections when categorising an activity
- An introductory video on how to use the digital tool
- Adding definitions of key terms and representations of key psychoeducational elements e.g., cycles of low mood and activation to support understanding
- Additional signposting embedded within the digital tool e.g., telephone numbers of support services.
- Opportunities for visual customisation—e.g., different colour schemes (including dark mode) and font sizes

Alongside these specific objectives, the feedback points towards a broader F appetite to make the digital tool multi-platform so that it can be accessed not only via a website but also potentially more immediately via a smartphone/tablet app. Also, and although this was not something directly mentioned in the feedback, we need to consider how we can best promote the tool to future participants in COMBAT BA. This includes identifying with our young people experts-by-experience the optimal platforms to advertise ComBAT Digital (e.g., TikTok, Instagram, etc), and speaking to professionals, commissioners and trainers about how to best drive adoption of the intervention by mental health support staff in schools, colleges and other community settings.

As well as determining the viability and acceptability of different digital platforms and modes of promoting the tool, a future feasibility study needs to assess the digital tool's acceptability across a broad demographic (including those who disengage earlier from support or who may be less digitally confident). Such a study can then pave the way for a fully powered trial testing the digital tool's clinical and cost-effectiveness in the context of the parent ComBAT BA programme, and exploring the specific mechanisms of change.

## 5 Conclusions

Overall, the young people interviewed had a positive experience with the new digital tool. They perceived it to be a useful and accessible resource that could support their mental health journey, both during and after therapy. The digital tool's features for planning, reflection, and visualizing progress were particularly valued. The feedback also highlighted several key areas for improvement. The most significant of these is the need to develop the digital tool into a downloadable app to improve accessibility and user experience. The inclusion of reminders, in-app guidance, customization options, and a journalling feature would also enhance engagement and the therapeutic value of the digital tool. Future evaluation needs to focus on generating evidence for the digital tool's clinical and cost-effectiveness.

## Data Availability

The raw data supporting the conclusions of this article will be made available by the authors, without undue reservation.
