# Supplementary material for: Usability testing of a digital tool to support behavioural activation for depression with young people aged 12 to 18
Source: Front Child Adolesc Psychiatry. 2026 May 21;5:1799929. doi: 10.3389/frcha.2026.1799929 (PMC13233449; doi:10.3389/frcha.2026.1799929)
Supplement: Supplementary file 1 [file Table1.docx]

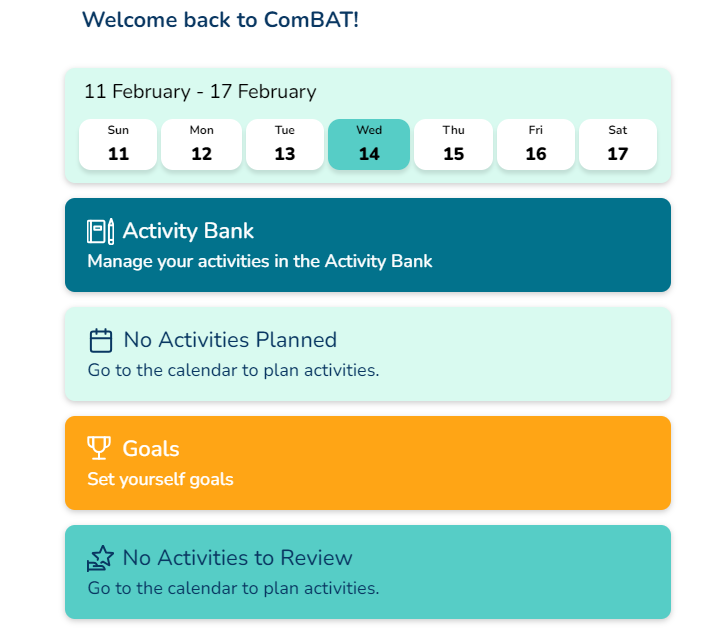


This is a screenshot of the home page of the ComBAT digital app which appears when the participant logs in. This links to all the key components of BA which the app supports.


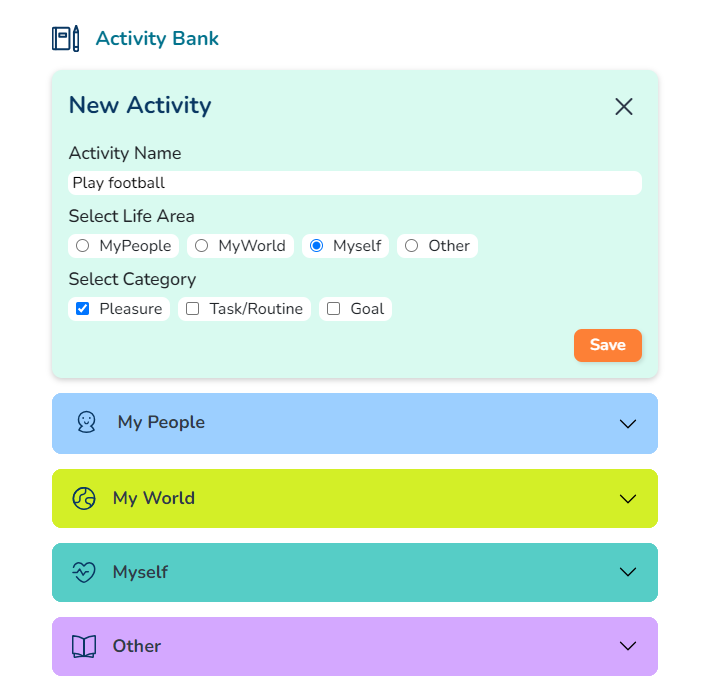


This is a screenshot of the ‘Activity Bank’ section of the ComBAT app. Here the young person can build a bank or pool of activities, mapping onto different areas of importance in their life ‘My People, My World’ etc. and fulfilling different functions ‘Pleasure, Task/Routine’ etc.


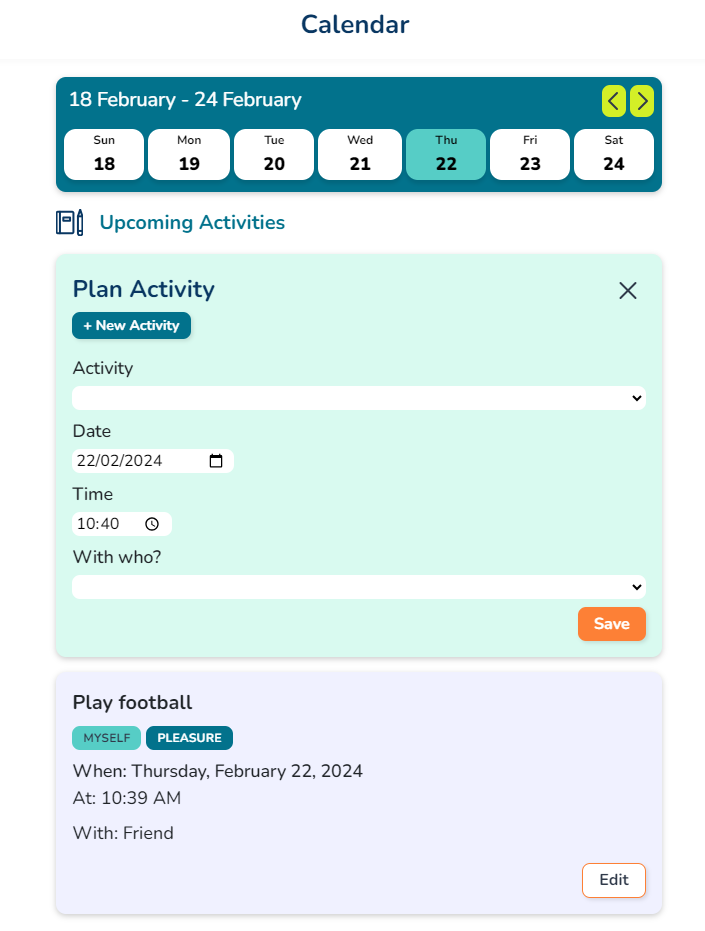


This is a screenshot of the calendar element of the app, where the young person can schedule activities drawn from their ’Activity Bank’.


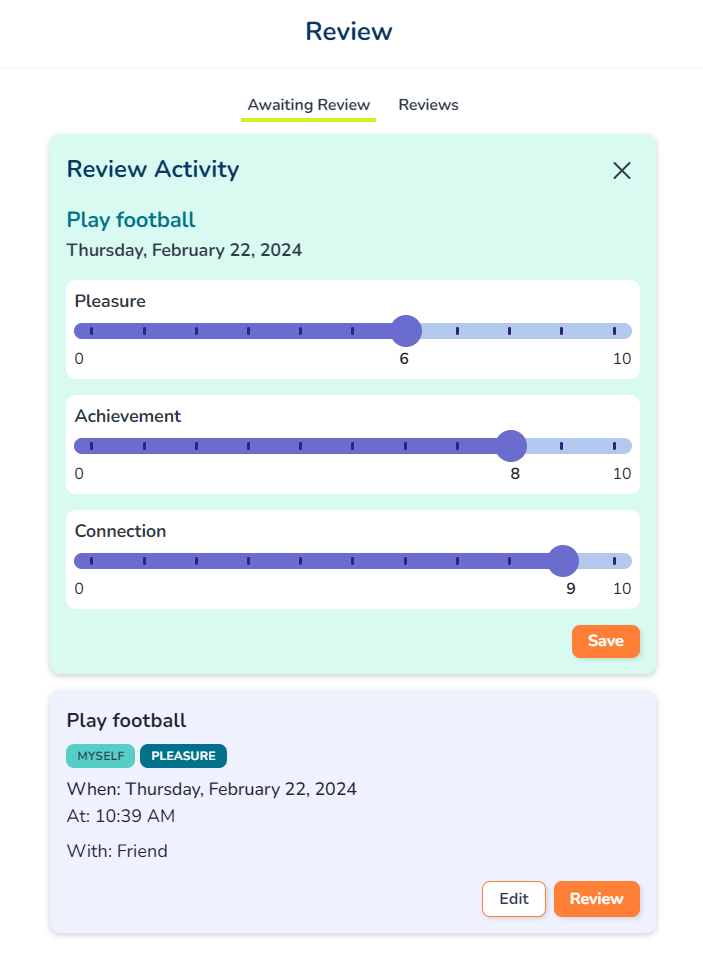


This screenshot shows the section of the app where the young person can review and rate the activities they have undertaken, scoring them according to the emotional rewards which they provided (PAC scores).


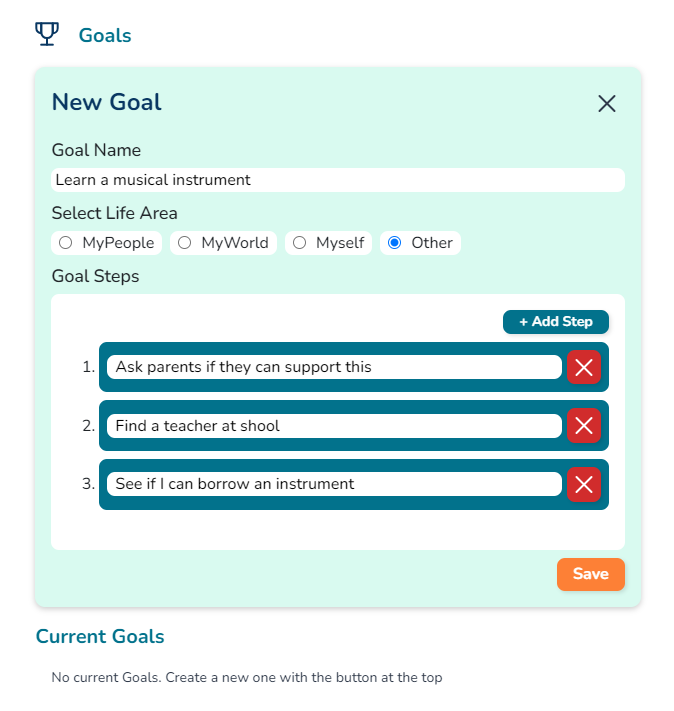


This screenshot shows the section of the app where the young people can set themselves longer-term goals and break these down into a number of achievable steps which they can tick-off once completed.


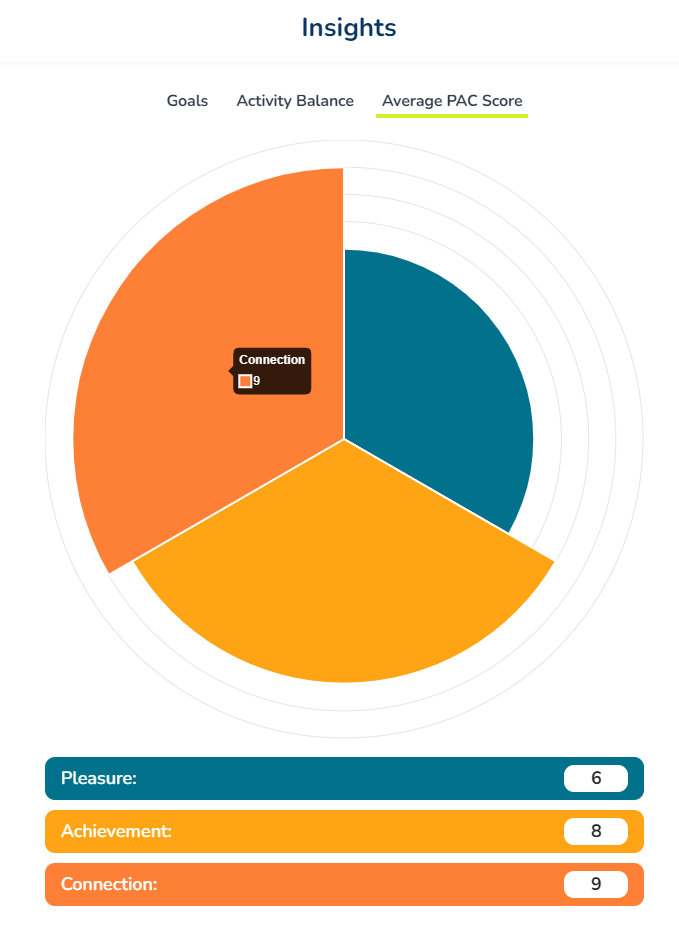


The app also includes an ‘Insights’ section which presents the young person with some of the key data around their engagement with BA in an visual and immediately accessible format.
